# Supplementary material for: Multiple contexts and frequencies aggregation network for deepfake detection
Source: PLoS One. 2026 Jan 29;21(1):e0337409. doi: 10.1371/journal.pone.0337409 (PMC12854465; doi:10.1371/journal.pone.0337409)
Supplement: Attachment — Submitted filename: plosone response.pdf [file pone.0337409.s001.pdf]

Reviewer #1

Basic Reporting

Comment 1.1: Expand the literature review by incorporating recent works, including the suggested references.

Response:

We have expanded the literature review by discussing more recent studies on deepfake detection, including the references suggested by the reviewer. The revised section now provides a broader context for our work and highlights its novelty.

Comment 1.2: Enhance figure captions, especially Figure 3, to better describe the architectural contributions of MKA and MFA.

Response:

Thank you for your suggestion. We have revised the Figure 3 caption to better describe the roles of the Multi-Kernel Aggregation (MKA) and Multi-Frequency Aggregation (MFA) blocks. Specifically, we now clarify that MKA acts as a token mixer, utilizing multi-dilation depthwise convolutions to extract multi-scale spatial features, improving sensitivity to subtle manipulations. Meanwhile, MFA functions as a channel mixer, incorporating frequency-aware feature decomposition and gating mechanisms to enhance forgery artifact extraction. Additionally, we refined the descriptions of the EfficientNet and ConvNeXt blocks, highlighting their roles as comparative baselines. These modifications improve clarity and reinforce MkfaNet's advantages in deepfake detection.

Experimental Design

Comment 1.3: Provide more details about the datasets, including specific forgery techniques and environmental conditions.

Response:

Thank you for your valuable suggestion. We have revised the Experimental Setup section to provide a more detailed description of each dataset, explicitly stating the forgery techniques (e.g., autoencoder-based Deepfake generation, expression reenactment, face-swapping, and neural texture synthesis) and their corresponding manipulation methods. Additionally, we have included information on environmental variations (e.g., lighting conditions, compression artifacts, and occlusions) that may influence model performance. Furthermore, we clarified how certain datasets, such as CelebDF-v2 and DF-1.0, introduce additional challenges due to reduced visual artifacts and real-world perturbations. To ensure fairness, we have also highlighted the use of DeepfakeBench's pre-processing workflow, maintaining consistent face cropping, resolution standardization (256×256), and normalization procedures across all datasets. These improvements provide a clearer understanding of the dataset diversity and its impact on the robustness of our proposed method.

Comment 1.4: Include specific hyperparameter settings for MKA and MFA to facilitate reproducibility.

Response:

Thank you for your suggestion. We have provided detailed hyperparameter settings for MKA

and MFA in Figure 3, where we explicitly specify the channel division ratios, kernel sizes, and dilation rates used in the MKA module, as well as the design of the MFA module. Additionally, Table 1 presents the complete architectural configurations of our two model variants (MkfaNet-Tiny and MkfaNet-Small), detailing the embedding dimensions, number of Mkfa blocks, MLP ratios, and FLOPs at each stage.

Validity of the Findings

Comment 1.5: Discuss potential limitations, such as scalability to larger datasets or performance on unseen forgery techniques.

Response:

Thank you for your suggestion. We have revised the Conclusion section to include a discussion on the limitations of our proposed MkfaNet. Specifically, we highlight two key challenges: (1) scalability to larger and more diverse datasets, as real-world deepfake videos often exhibit greater variability in quality and manipulation techniques, and (2) generalization to novel deepfake generation methods, as emerging techniques may introduce more sophisticated forgery patterns that require adaptive detection mechanisms. By acknowledging these limitations, we provide a clearer perspective on areas for future improvement, including expanding MkfaNet to larger datasets and developing more dynamic feature extraction strategies to enhance robustness against evolving forgery techniques.

Comment 1.6: Provide a detailed analysis of misclassifications to identify patterns and propose strategies for improvement.

Response:

The confusion matrix in Table 4 indicates that the model misclassified 459 fake images as real (FN) and 181 real images as fake (FP) on the CelebDF-v1 dataset. As shown in Figure 5, FP cases mainly occur due to compression artifacts, occlusions, and extreme facial expressions, which introduce inconsistencies similar to Deepfake manipulations. FN cases arise from high-quality Deepfakes that lack visible artifacts or primarily modify low-frequency components while preserving high-frequency details, making them harder to detect. To improve performance, multi-task learning could incorporate forgery localization and frequency-aware supervision to enhance subtle manipulation detection, while adaptive feature fusion can better handle low-frequency forgeries. Additionally, data augmentation with compression artifacts, occlusions, and diverse Deepfake techniques can improve generalization and reduce misclassification rates, further strengthening the model’s robustness in real-world Deepfake detection tasks.

Reviewer #2

Abstract

Comment 2.1: Add numerical values in the abstract to demonstrate the system's efficiency.

Response:

Thank you for your suggestion. We have revised the abstract to include numerical results demonstrating the efficiency of our proposed MkfaNet. Specifically, we report the AUC scores from Table 2, where MkfaNet-S achieves an AUC of 0.9591 in within-domain evaluations and

0.7963 in cross-domain evaluations, outperforming multiple state-of-the-art methods while maintaining high computational efficiency. These numerical results provide a clearer representation of our model's effectiveness in deepfake detection.

Introduction

Comment 2.2: Include a paragraph at the end of the introduction outlining the structure of the paper.

Response:

Thank you for your suggestion. We have revised the Introduction section by adding a paragraph at the end that provides an overview of the paper structure. The revised introduction now clearly outlines the organization of the paper as follows: Section 2 discusses related work, providing an overview of deepfake detection methods and their limitations. Section 3 presents our proposed MkfaNet, detailing its architecture and core components. Section 4 describes the experimental setup and results, including dataset details, evaluation metrics, and comparative analysis. Finally, Section 5 concludes the paper, summarizing key findings and suggesting future research directions. This addition improves the readability and coherence of the paper.

Related Work

Comment 2.3: Clearly state the major drawbacks of previous related work instead of mentioning them within individual studies.

Response:

Thank you for your valuable suggestion. We have revised Section 2 (Related Work) to explicitly discuss the key drawbacks of previous deepfake detection approaches. Instead of only referring to limitations while listing individual studies, we have added a dedicated paragraph summarizing the main challenges. Specifically, we highlight (1) the reliance on manually designed spatial or frequency features, which may not generalize well across datasets, (2) the limitations of using traditional deep learning backbones, which may lose critical local forgery details due to their hierarchical feature extraction process, and (3) the difficulty of detecting high-quality deepfakes, as newer techniques create increasingly realistic facial textures and seamless blending. These points emphasize the necessity of our proposed MkfaNet, which integrates Multi-Kernel Aggregation (MKA) and Multi-Frequency Aggregation (MFA) modules to address these limitations and improve deepfake detection robustness.

Methodology

Comment 2.4: Explain the steps of the proposed algorithm in an algorithmic manner.

Response:

Thank you for your suggestion. While we understand the importance of presenting an algorithmic description, our proposed MkfaNet is a backbone architecture rather than a sequential algorithm, making it less suitable for a step-by-step procedural explanation. Instead of a strict algorithmic format, we have structured our methodology section to clearly outline the key components and their roles in the detection process. Specifically, we have already detailed how the Multi-Kernel Aggregator (MKA) captures spatial features and how the Multi-Frequency Aggregator (MFA) extracts frequency information, along with the

classification process. This structured explanation effectively conveys our model's operation without requiring an explicit algorithm format. Instead of an explicit algorithm format, we have further clarified the detection pipeline using Figure 2, which visually represents the hierarchical four-stage architecture of MkfaNet. Each stage consists of an embedding stem, Multi-Kernel Aggregator (MKA), and Multi-Frequency Aggregator (MFA) blocks, which extract spatial and frequency-aware features progressively. The final classification head outputs a binary prediction (real or fake). This structured representation effectively conveys our model’s detection process without requiring an explicit algorithmic format.

Conclusion

Comment 2.5: Build conclusions based on numerical values of the results and clarify future work.

Response:

Thank you for your suggestion. We have revised the Conclusion section to provide a clearer discussion of future work and support our findings with numerical performance results. Specifically, we now highlight two key directions for future improvements: (1) expanding MkfaNet to handle more diverse and large-scale datasets, improving robustness against real-world deepfake variations, and (2) enhancing adaptability to novel forgery techniques, ensuring the model remains effective as deepfake generation methods evolve. Additionally, we have included the numerical performance results from Table 2, explicitly stating that MkfaNet-S achieves an AUC of 0.9591 in within-domain evaluations and 0.7963 in cross-domain evaluations, demonstrating its superior detection performance. These revisions ensure a more concrete and well-supported conclusion.

Reviewer #3

Comment 3.1: The approach to incorporating both spatial and frequency priors is innovative and aligns well with the increasing need for multi-scale feature extraction in deepfake detection. However, the paper could benefit from a clearer comparison of MkfaNet with existing methods that similarly combine spatial and frequency-based approaches.

Response:

Thank you for your valuable feedback. Our work introduces MkfaNet as a novel backbone that uniquely integrates spatial and frequency priors through the Multi-Kernel Aggregator (MKA) and Multi-Frequency Aggregator (MFA) modules. Unlike existing methods that utilize separate spatial or frequency-based feature extraction techniques, MkfaNet jointly optimizes both domains in a unified backbone, leading to improved generalization across diverse deepfake datasets.

To provide a clearer comparison, we evaluate MkfaNet across three different types of deepfake detection methods in DeepfakeBench, as detailed in Table 2, demonstrating its effectiveness in multiple detection paradigms.

Comment 3.2: While the introduction effectively motivates the problem and the solution, more detailed discussions on the challenges posed by specific types of deepfakes (e.g., face

swapping, facial expression modification) could strengthen the context.

Response:

Thank you for your valuable suggestion. We have already discussed the general challenges of deepfake detection in the Introduction section, but we acknowledge the importance of elaborating on the challenges posed by specific types of deepfakes, such as face swapping, facial expression modification, and identity manipulation.

To strengthen the context, we emphasize that face swapping techniques often introduce blending artifacts and inconsistencies in identity-related features, making them difficult to detect without spatial-aware feature extraction. Similarly, facial expression modification creates subtle yet unnatural deformations, which are better captured through frequency-based analysis. These challenges further justify our dual-domain approach in MkfaNet, which effectively captures spatial inconsistencies via MKA and frequency discrepancies via MFA to enhance deepfake detection across different manipulation types.

Comment 3.3: The description of the Multi-Kernel Aggregator (MKA) is insightful, but it would be helpful to include more experimental validation regarding the effectiveness of different dilation rates in various scenarios.

Response:

To validate the effectiveness of different dilation rates in the Multi-Kernel Aggregator (MKA) module, we conducted ablation experiments and reported the results in Table 5. The table compares different channel allocation ratios for dilation rates (c1:c2:c3), showing that the 1:3:4 setting achieves the highest AUC, demonstrating its effectiveness in capturing multi-scale spatial features. These results confirm that balancing receptive fields with appropriate dilation rates enhances Deepfake detection performance, supporting our design choice for MKA.

Comment 3.4: The Multi-Frequency Aggregator (MFA) module is well explained, but an analysis of how it adapts to different frequency bands would clarify its robustness and potential limitations in handling varying image qualities or noise levels.

Response:

Thank you for your suggestion. The Multi-Frequency Aggregator (MFA) module is designed to process different frequency bands through depthwise convolutions and gating mechanisms, allowing the model to adapt to diverse image qualities and noise levels. Table 4 presents an ablation study demonstrating that adding multi-frequency (MF) processing improves the AUC to 0.9176, confirming the effectiveness of MFA in handling frequency-based artifacts.

Comment 3.5: The visual results presented in Figure 1 demonstrate the advantages of MkfaNet in capturing high-frequency anomalies. However, it would be beneficial to include more diverse examples of real vs. fake images to better showcase the method's generalization capabilities.

Response:

Thank you for your suggestion. Figure 1 already provides diverse examples of real and fake faces from two different datasets (FFD and CDFv1), ensuring a fair evaluation of MkfaNet’s ability to capture high-frequency anomalies across different forgery types.

Additionally, MkfaNet's generalization capability is further validated in Table 2, where cross-domain evaluations across six different datasets demonstrate its robustness against various deepfake manipulations. Given that these quantitative results already support MkfaNet's ability to generalize well, we believe that the current visual examples sufficiently represent the model's effectiveness. However, we appreciate the reviewer's insight and will consider expanding the visual comparisons in future work to further illustrate MkfaNet's adaptability.

Comment 3.6: The paper mentions the efficiency of MkfaNet in terms of parameter usage. A deeper dive into the model's computational efficiency and comparison with state-of-the-art methods would provide further insight into its practical application in real-time detection systems.

Response:

Thank you for your valuable suggestion. To provide a more comprehensive evaluation of MkfaNet's computational efficiency, we have included its FLOPs in Table 1, alongside its parameter count. These results demonstrate that MkfaNet achieves an efficient balance between computational cost and detection performance.

Comment 3.7: The section on model training could be enhanced by providing more details on the dataset splits and how the cross-domain evaluations were conducted. This would help readers understand the robustness of the model in diverse settings.

Response:

Thank you for your suggestion. To improve clarity, we have expanded the dataset descriptions in the Experimental Setup section. Following DeepfakeBench, we conduct evaluations on seven deepfake detection datasets, using FF++ (c23) for training while the remaining datasets serve as test sets for cross-domain evaluation, as shown in Table 2.

Each dataset presents unique challenges, such as the improved boundary artifacts in CelebDF-v2, the diverse manipulation techniques in DFDC, and the real-world perturbations in DF-1.0, making them suitable for assessing generalization. To ensure consistency, we adopt DeepfakeBench's preprocessing workflow and use a fixed 256×256 resolution for cropped face images.

Comment 3.8: The architecture of MkfaNet is described clearly, but a detailed analysis of the trade-offs between performance and model complexity would help justify the design choices, especially for applications with limited resources.

Response:

Thank you for your valuable suggestion. To better illustrate the balance between performance and model complexity, we provide two variants of MkfaNet (MkfaNet-T and MkfaNet-S) with different parameter sizes and FLOPs, as detailed in Table 1.

Our results demonstrate that MkfaNet-T achieves competitive detection accuracy with significantly lower computational cost, making it suitable for resource-constrained scenarios. Meanwhile, MkfaNet-S, with a higher parameter count, provides superior performance but requires more computational resources. This trade-off allows users to choose the appropriate variant based on their application requirements, ensuring flexibility for both efficiency-sensitive and high-performance tasks.

Comment 3.9: The conclusion is promising, but it would be useful to discuss potential areas for future work, such as the integration of MkfaNet with other AI-driven detection systems or its adaptation to other types of synthetic media beyond deepfakes.

Response:

Thank you for your suggestion. In the revised manuscript, we have expanded the Conclusion section to discuss future directions. Specifically, we acknowledge the scalability challenge when applying MkfaNet to large and diverse datasets and the difficulty in generalizing to emerging deepfake techniques. Future work will focus on enhancing model robustness through larger-scale training and adaptive feature extraction. Additionally, we will explore MkfaNet's integration with multimodal detection systems and its extension to other types of synthetic media to further improve its applicability.

Comment 3.10: Improve the literature discussion on machine learning-based feature selection methods.

Response:

Thank you for your suggestion. We have expanded the Related Work section by incorporating discussions on semi-supervised learning and feature selection techniques in deepfake detection, along with additional citations to recent machine learning-based methods to strengthen the contextual foundation of our study.

Reviewer #4

Novelty and Contributions

Comment 4.1: Clearly differentiate MkfaNet from existing methods and highlight its contributions.

Response:

Thank you for your insightful comment. Our primary contribution is the introduction of MkfaNet as a novel backbone architecture specifically designed for deepfake detection. The key difference between our approach and existing methods lies in the backbone design, which integrates Multi-Kernel Aggregator (MKA) and Multi-Frequency Aggregator (MFA) modules to enhance spatial and frequency-aware feature learning.

To ensure a comprehensive comparison, we consider three types of detectors in DeepfakeBench, as detailed in Table 2, demonstrating that MkfaNet achieves superior performance across different detection paradigms. These results further validate the effectiveness of our proposed backbone in deepfake detection.

Methodology

Comment 4.2: Methodology Details: The descriptions of the proposed modules (MKA and MFA) need to be more comprehensive, including the rationale behind design choices, computational complexity, and implementation details to improve reproducibility. The visualization of the proposed architecture should be refined for better clarity.

Response:

Thank you for your suggestion. We have already provided detailed descriptions of the Multi-

Kernel Aggregator (MKA) and Multi-Frequency Aggregator (MFA) modules in the Methodology section, explaining their roles in capturing spatial and frequency-aware features. The rationale behind their design is to enhance the detection of deepfake artifacts by adaptively learning spatial and frequency-based inconsistencies.

To address computational complexity, we have included the parameter count and FLOPs for different MkfaNet variants in Table 1, demonstrating the trade-off between efficiency and performance. Additionally, Figure 3 (subfigures a and b) illustrates the detailed structures and hyperparameters of MKA and MFA, further clarifying their internal mechanisms. These additions ensure transparency and reproducibility in our methodology.

Comment 4.3: Experimental Analysis: The evaluation lacks critical aspects such as failure case analysis, false positive/negative rates, and statistical significance tests, which are essential to validate the model's robustness and practical applicability.

Response:

To address concerns about robustness and practical applicability, we analyze failure cases and false positive/negative rates using Table 4, which highlights that the model misclassified 459 fake images as real (FN) and 181 real images as fake (FP) on the CelebDF-v1 dataset. Furthermore, Table 2 demonstrates that MkfaNet consistently outperforms state-of-the-art Deepfake detection models across multiple datasets in both within-domain and cross-domain evaluations, indicating strong generalization ability.

Computational Efficiency

Comment 4.4: Provide FLOPs and inference time for practical deployment feasibility.

Response:

Thank you for your suggestion. To better illustrate the computational efficiency of MkfaNet, we have included the FLOPs for two different model sizes in Table 1, representing the trade-off between model complexity and efficiency. These results demonstrate that MkfaNet maintains a balance between high detection performance and computational feasibility, making it suitable for real-world applications.
