# Supplementary material for: Multiple contexts and frequencies aggregation network for deepfake detection
Source: PLoS One. 2026 Jan 29;21(1):e0337409. doi: 10.1371/journal.pone.0337409 (PMC12854465; doi:10.1371/journal.pone.0337409)
Supplement: Attachment — Submitted filename: AuthorResponse.pdf [file pone.0337409.s002.pdf]

Response to Reviewer #5

Comment 1: The authors are advised to consider integrating a brief comparative table summarizing key differences between their method and related works to further clarify your unique contributions.

Response:

We thank the reviewer for this valuable suggestion. To better highlight the distinguishing characteristics of our proposed MkfaNet, we have added a comparative table (Table 1) in the section 3.4. This table summarizes the differences among representative backbone models in terms of feature extraction strategy, spatial aggregation, and frequency aggregation capabilities. Unlike prior methods such as XceptionNet, EfficientNet, and ConvNeXt, which lack either spatial or frequency modeling modules, our MkfaNet explicitly integrates both through the Multi-Kernel Aggregator (MKA) and Multi-Frequency Aggregator (MFA), enabling more effective representation of subtle local and frequency-domain forgery cues.

Comment 2: The revised Figure 3 caption now clearly explains the roles of the Multi-Kernel Aggregator (MKA) and Multi-Frequency Aggregator (MFA), which aids reader comprehension. The authors are advised to expand the range of visual examples (e.g., additional real vs. fake comparisons), which would enhance the demonstration of the authors' model's generalization capabilities.

Response:

We thank the reviewer for this insightful suggestion. To provide a more comprehensive view of the model's behavior, we have revised Figure 6 to include four representative prediction categories: True Positive (TP), True Negative (TN), False Positive (FP), and False Negative (FN). Each row presents visual examples along with the predicted confidence scores. These additions better illustrate how the proposed MkfaNet handles both successful and failure cases across a range of visual conditions such as occlusion, compression artifacts, and subtle expression variations. By expanding the range of visual outcomes, we aim to provide a clearer and more balanced understanding of our model's strengths and current limitations in real-world scenarios.

Comment 3: The authors are advised to further include details on training procedures (such as specific data splits, augmentation strategies, and training durations). This would be beneficial for readers seeking to replicate the experiments..

Response:

Thank you for your helpful suggestion. We have revised the Implementation Details section to provide more comprehensive training information. Specifically, we clarify that all data splits strictly follow the DeepfakeBench protocol. Cropped face images are resized to 256 × 256. Classical CNN-based detectors are trained using the Adam optimizer (learning rate = 2e-4, batch size = 32), while detectors with modern backbones (including MkfaNet) are trained using the AdamW optimizer (learning rate = 5e-4, batch size = 256). All models are trained for 50 epochs, with the best-performing checkpoint selected based on validation results. We also utilize ImageNet-1K pretrained weights for backbone initialization where applicable. These clarifications aim to enhance the reproducibility and transparency of our experimental

setup.

Comment 4: Further analyses: The revisions include misclassification analyses and a discussion of limitations such as scalability and generalization to unseen forgery techniques. This discussion is appreciated and adds balance to the manuscript. To further enhance the robustness of the authors' claims, it is recommended to incorporate statistical significance tests (e.g., confidence intervals or p-values) for the performance metrics.

Response:

We sincerely thank the reviewer for acknowledging our efforts in misclassification analysis and the discussion of limitations. We fully agree that incorporating statistical significance tests would further enhance the rigor of performance evaluation. In response to the reviewer's suggestion, we have now included confidence intervals for the performance metrics and updated the results in Table 3 accordingly. These confidence intervals were calculated based on the results from multiple runs and provide a more robust evaluation of the detectors' performance across different datasets. We believe this addition enhances the transparency and reliability of our findings.

Comment 5: Methodology: Although the authors noted that a procedural algorithmic description is not directly applicable to a backbone architecture, a flowchart summarizing the detection pipeline could serve as an alternative means to assist readers in understanding the overall process.

Response:

We appreciate the reviewer's suggestion. We agree that a clear visual representation of the detection pipeline can significantly improve reader understanding. Accordingly, we emphasize that Figure 2 in the manuscript already serves as a flowchart for our detection pipeline. It illustrates the full process from input face image to final prediction, including all intermediate components such as the embedding stem, Multi-Kernel Aggregator (MKA), Multi-Frequency Aggregator (MFA), and the final fully connected classification layer. We have slightly revised the caption of Figure 2 to clarify its role as a complete architectural overview.

Comment 6: Computational Efficiency: The inclusion of FLOPs, parameter counts, and a discussion on computational trade-offs is informative. An expanded discussion on how these metrics translate to real-world deployment, including inference times and resource requirements in various scenarios, would further strengthen the paper.

Response:

Thank you for the thoughtful suggestion. We agree that connecting model complexity to real-world deployment is important. To address this, we have added a discussion on practical inference performance. Specifically, MkfaNet-Tiny and MkfaNet-Small contain 5.2M/1.5G and 19.8M/6.7G parameters/FLOPs, respectively. We tested their inference performance on an RTX 4090 GPU with a batch size of 480 images, and the average inference time was approximately 22 ms for MkfaNet-Tiny and 98 ms for MkfaNet-Small. These results indicate that our model variants are capable of efficient high-throughput deployment on modern GPU hardware.
