# Supplementary material for: Multiple contexts and frequencies aggregation network for deepfake detection
Source: PLoS One. 2026 Jan 29;21(1):e0337409. doi: 10.1371/journal.pone.0337409 (PMC12854465; doi:10.1371/journal.pone.0337409)
Supplement: Attachment — Submitted filename: response.pdf [file pone.0337409.s003.pdf]

Author Response on Submission PONE-D-24-45189R3

We would like to express our sincere gratitude to the Editor and the Reviewers for their time, constructive feedback, and positive evaluation of our manuscript. We are pleased to note that Reviewers #3 and #5 have confirmed that all prior comments have been fully addressed, and that the work is technically sound, clearly written, and meets PLOS ONE’s publication standards. Reviewer #2 did not provide additional comments in this round.

In this revision, we have carefully fulfilled the editorial request concerning code and data availability to ensure full compliance with the PLOS ONE Materials and Software Sharing Policy. All author-generated code and configuration files used in this study have been made available at: https://github.com/GGshawn/MkfaNet

This repository contains the complete deepfake detection framework, including baseline detectors and our proposed MkfaNet backbone, along with dataset download and preprocessing tools, training and evaluation scripts, and pretrained checkpoints, enabling full reproducibility. All datasets used in this work are publicly accessible, and repository documentation provides clear download and preprocessing instructions.

No further textual changes were required based on reviewer feedback. We once again thank the Editor and Reviewers for their thoughtful evaluation and for supporting the publication of our work.
